# EF1α and RPL13a represent normalization genes suitable for RT-qPCR analysis of bone marrow derived mesenchymal stem cells

**DOI:** 10.1186/1471-2199-11-61

**Published:** 2010-08-17

**Authors:** Kevin M Curtis, Lourdes A Gomez, Carmen Rios, Elisa Garbayo, Ami P Raval, Miguel A Perez-Pinzon, Paul C Schiller

**Affiliations:** 1 Geriatric Research, Education, and Clinical Center, Veterans Affairs Medical Center and The Geriatrics Institute, 1201 NW 16th Street, Miami, Florida 33125 USA; 2 South Florida Veterans Affairs Foundation for Research and Education, Inc., 1201 NW 16th Street, Miami, Florida 33125 USA; 3 Department of Biochemistry & Molecular Biology, University of Miami Miller School of Medicine, 1011 NW 15th Street, Miami, Florida 33101 USA; 4 Department of Neurology, University of Miami Miller School of Medicine, 1120 NW 14th Street, Miami, Florida 33136 USA; 5 Cerebral Vascular Disease Research Center, University of Miami Miller School of Medicine, 1120 NW 14th Street, Miami, Florida 33136 USA; 6 Department of Medicine, University of Miami Miller School of Medicine, 1500 NW 12th Avenue, Miami, Florida, 33136 USA; 7 Neuroscience Program, University of Miami Miller School of Medicine, 1120 NW 14th Street, Miami, Florida, 33136 USA

## Abstract

**Background:**

RT-qPCR analysis is a widely used method for the analysis of mRNA expression throughout the field of mesenchymal stromal cell (MSC) research. Comparison between MSC studies, both *in vitro *and *in vivo*, are challenging due to the varied methods of RT-qPCR data normalization and analysis. Therefore, this study focuses on putative housekeeping genes for the normalization of RT-qPCR data between heterogeneous commercially available human MSC, compared with more homogeneous populations of MSC such as MIAMI and RS-1 cells.

**Results:**

Eight genes including; *ACTB, B2M, EF1α, GAPDH, RPL13a, YWHAZ, UBC* and *HPRT1* were tested as possible housekeeping genes based on their expression level and variability. *EF1α *and *RPL13a *were validated for RT-qPCR analysis of MIAMI cells during expansion in varied oxygen tensions, endothelial differentiation, neural precursor enrichment, and during the comparison with RS-1 cells and commercially available MSC. *RPL13a *and *YWHAZ *were validated as normalization genes for the cross-species analysis of MIAMI cells in an animal model of focal ischemia. *GAPDH*, which is one of the most common housekeeping genes used for the normalization of RT-qPCR data in the field of MSC research, was found to have the highest variability and deemed not suitable for normalization of RT-qPCR data.

**Conclusions:**

In order to make comparisons between heterogeneous MSC populations, as well as adult stem cell like MSC which are used in different laboratories throughout the world, it is important to have a standardized, reproducible set of housekeeping genes for RT-qPCR analysis. In this study we demonstrate that *EF1α*, *RPL13a *and *YWHAZ *are suitable genes for the RT-qPCR analysis and comparison of several sources of human MSC during *in vitro *characterization and differentiation as well as in an *ex vivo* animal model of global cerebral ischemia. This will allow for the comparative RT-qPCR analysis of multiple MSC populations with the goal of future use in animal models of disease as well as tissue repair.

## Background

Human bone marrow-derived multipotent mesenchymal stromal cells (MSC) represent a unique but heterogeneous population of progenitor cells (adult stem cells) with self-renewal properties and multilineage differentiation potential [1]. Various isolation, selection, and culture conditions have been used (review [2]) in order to develop more homogeneous populations of human MSC such as MIAMI cells [3], MAPC, MASC [4], SSEA-4+ MSC [5], CD133+ Selected MSC [6], and RS-1 cells [7]. These sub-populations of MSC are characterized by increased self-renewal potential and the ability to differentiate not only into mature cells found in mesodermal-derived tissues (SSEA-4+ MSC), but also in ectodermal-and endodermal-derived tissues (MIAMI cells, MAPC, MACS, CD133+ Selected MSC).

The *in vitro, ex vivo*, and *in vivo *characterization of MSC requires the analysis of gene expression profiles in order to understand their underlying mechanisms of self-renewal during long term expansion, differentiation into all three germinal lineages, as well as their tissue repair properties in pre-clinical models of disease. Quantitative real time RT-PCR (RT-qPCR) is often used as a tool to determine the relative change of a target genes mRNA expression, which is normalized against a highly expressed and stable reference gene. Due to its affordability, ease of use, and reproducibility, RT-qPCR is used widely throughout the field of MSC research. However, the validity of gene expression data determined by RT-qPCR is dependent on the optimal selection of at least two or more reference genes for normalization, characterized by high expression levels and low expression variability [8,9].

The purpose of this study was to validate at least two reference genes suitable for the normalization of RT-qPCR gene expression data in MSC such as MIAMI cells under various conditions including: (1) low and ambient oxygen tension (pO_2_), (2) expansion and or differentiation, (3) *ex vivo *or *in vivo *animal disease models, (4) determination of consistent gene expression profiles across several MSC subpopulation and preparations. Due to the varied nature of gene expression, we selected 8 genes involved in different cellular functions and widely employed as normalization genes in the literature. These genes include: transcript translation (*EF1α*, *RPL13a*), cell motility/cytoskeleton (*ACTB*), immune response/binds MHC class I (*B2M*), metabolism/glycolysis (*GAPDH*), nucleotide salvaging/purine synthesis (*HPRT1*), signal transduction (*YWHAZ*), and protein degradation (*UBC*) (Table# 1, 2). A previous study also showed that, *UBC*, *RPL13a*, and *YWHAZ *are 3 suitable reference genes for RT-qPCR analysis of whole bone marrow aspirates [8].

**Table 1:** Review of normalization "housekeeping" genes used for RT-qPCR analysis of mesenchymal stromal cells

RT-qPCR		
**Gene**	**Full Name**	**Reference**
*SMa-actin*	alpha-2 smooth muscle aorta actin	Ross et al 2006[26]
*ACTB*	β-actin	Dickhut et al 2009 [27], Murthy et al 2008 [28]
*GAPDH*	Glyceraldehyde-3-phosphate dehydrogenase	Block et al 2009 [29], Gang et al 2009 [30], *Lu et al2009 [31], Leonardi et al 2009 [32], Schneider et al 2009 [33], Tan et al 2009 [34], Corcoran et al 2008 [35],
*B2M*	β2-microglobulin	Briquet et al 2009 [36]

**Semi-quantitative or normal PCR**		

**Gene**	**Full Name**	**Reference**
*EF1α*	Eukaryotic translational elongation factor 1 alpha	D'Ippolito et al 2006 [15,37,38], D'Ippolito et al 2004 [3]
*ACTB*	β-actin	Riekstina et al 2009 [39], Gang et al 2007 [5], Shim et al 2004 [40]
*GAPDH*	Glyceraldehyde-3-phosphate dehydrogenase	Drost et al 2009 [41], Tang et al 2008 [42], Greco et al 2008 [43], Trzaska et al 2008 [44], Betrami et al 2007 [4], Greco et al 2007 [45], Tatard et al 2007 [17], Trzaska et al 2007 [46], Muguruma et al 2003 [47]
*B2M*	β2-microglobulin	Pozzobon et al 2009 [6], Martinez et al 2007 [48]

* used more than one normalization gene (*GAPDH *and *18 S RNA*)

Table#1: Literature searches were conducted through PubMed Central http://www.pubmed.com. Representative manuscripts were selected which used quantitative real time PCR (RT-qPCR) or semi-quantitative PCR analyses for the analysis of gene expression in human bone marrow derived adult stem cells or classical MSC to determine commonly used normalization "housekeeping" genes.

**Table 2:** Genes used for Real Time RT-qPCR analysis

Gene	Full Name	Accession Number	Sequence	Cellular Function	Reference
	***Normalization Genes***				
*ACTB*	Beta-actin	NM_001101	F = 5'-CTGGAACGGTGAAGGTGACA-3' R = 5'-AAGGGACTTCCTGTAACAATGCA-3'	Cell motility, structure, integrity	
*EF1α*	Eukaryotic translational elongation factor 1 alpha	NM_001402	F = 5'-AGGTGATTATCCTGAACCATCC-3' R = 5'-AAAGGTGGATAGTCTGAGAAGC-3'	Translation	
*B2M*	Beta-2-microglobulin	NM_004048	F = 5'-TGCTGTCTCCATGTTTGATGTATCT-3' R = 5'-TCTCTGCTCCCCACCTCTAAGT-3'	Immune Response: Binds MHC class 1	
*GAPDH*	Glyceraldehyde-3-phosphate dehydrogenase	NM_002046	F = 5'-TGCACCACCAACTGCTTAGC-3' R = 5'-GGCATGGACTGTGGTCATGAG-3'	Metabolism: Glycolysis	[8]
*HPRT1*	Hypoxanthine phosphoribosyltransferase 1	NM_000194	F = 5'-TGACACTGGCAAAACAATGCA-3' R = 5'-GGTCCTTTTCACCAGCAAGCT-3'	Nucleotide Salvaging: Purine synthesis	[8]
*RPL13a	Ribosomal protein L13a	NM_01242	F = 5'-CATAGGAAGCTGGGAGCAAG-3' R = 5'-GCCCTCCAATCAGTCTTCTG-3'	Translation	
*YWHAZ	Tyrosine 3-monooxygenase/tryptophan 5- monooxygenase activation	NM_003406 NM_145690	F = 5'- TGCTTGCATCCCACAGACTA-3' R = 5'- AGGCAGACAATGACAGACCA-3'	Signal Transduction	
*UBC*	Ubiquitin C	NM_021009	F = 5'-ATTTGGGTCGCGGTTCTTG-3' R = 5'-TGCCTTGACATTCTCGATGGT-3'	Protein Degradation	[8]
	***Target Genes***				
*CD31*	Platelet/endothelial cell adhesion molecule: PECAM1	NM_000442	F = 5'-AACAGTGTTGACATGAAGAGCC-3' R = 5'-TGTAAAACAGCACGTCATCCTT-3'		
*LTBP2	Latent transforming growth factor binding protein 2	NM_000428	F = 5'-GAGCCCAGCTGGAGTAGGA-3' R = 5'-AGCTTCTCTGAGTCTAGGGGG-3'		
*STC1	Stanniocalcin 1	NM_003155	F = 5'-AGGCAAGGCTGACTTCTCTG-3' R = 5'-AACTACTTGTCGCATTGGGG-3'		
*TSG6	Tumor necrosis factor, alpha- induced protein 6 (TNFAIP6)	NM_007115	F = 5'-TCACATTTCAGCCACTGCTC-3' R = 5'-TGATCATATCGTCAGTTGTAGTGAA-3'		
	***Rat Specific Normalization Genes***				
*rRPL13a*	Ribosomal protein L13a	NM_173340	F = 5'-GGCTGAAGCCTACCAGAAAG-3' R = 5'-CTTTGCCTTTTCCTTCCGTT-3'		
	***Rat Specific Target Genes***				
*rIGF1*	Insulin-like growth factor 1(somatomedin C), transcript variant 1-4	NM_178866 NM_001082477-9	F = 5'- GCTGAAGCCGTTCATTTAGC-3' R = 5'- GAGGAGGCCAAATTCAACAA-3'		
*rIGFBP3*	Insulin-like growth factor binding protein 3	NM_012588	F = 5'- CTCCATGTGCAGAGATGTCG-3' R = 5'- CTCTTTTGAAAGCTGCTCC-3'		
*rIGFBP5*	Insulin-like growth factor binding protein 5	NM_012817	F = 5'-AAGGAGACACTCCCCATTCC-3' R = 5'-TTCCCTTCTCTGTCCGTTCA-3'		

Table#2: Primer pairs preceded by (*) are human species specific primer pairs

Heterogeneous MSC and primitive more homogeneous population of bone marrow derived adult stem cell (MIAMI cells, RS-1 cells, MAPC etc.) are isolated from whole bone marrow aspirates and are a sub-fraction of the total bone marrow cell population. Reviewing the literature on bone marrow-derived adult stem cell research, *GAPDH*, *ACTB*, *B2M *and *EF1α *were found to be the most commonly used genes for normalization of RT-qPCR data (Table# 1). We validated the stability of the known whole bone marrow RT-qPCR reference genes *UBC*, *RPL13a*, and *YWHAZ *[8], as well as the previously mentioned genes used in MSC research. We analyzed the stability and expression profile of each reference gene in MIAMI cells using low oxygen tension (pO_2_), growth factor induced neural precursor enrichment, under growth factor stimulated endothelial differentiation conditions, and in an *ex vivo *rat hippocampal organotypic model of global cerebral ischemia. In addition, we compared the results in MIAMI cells to another population of bone marrow-derived adult stem cells, RS-1 cells [7] as well as commercially available MSC.

Adult stem cells such as bone marrow derived MIAMI cells are a promising source for cell therapy based approaches due to their immunomodulatory properties as well as their potential to differentiate into mature somatic tissues [10]. They are also not burdened by ethical restrictions or problems such as partial vs. full epigenetic reprogramming, tumorgenicity potential, nor due to controversial clinical functionality associated with embryonic stem cells (ESC) and induced pluripotent stem (iPS) cells [11,12]. Our study identified *EF1α *and *RPL13a *as ideal reference genes for RT-qPCR analysis of MSC. These results are important because they will allow for the valid, reproducible, and comparative analysis of gene expression data in an increasingly expanding area of MSC research, especially for future clinical use.

## Results

### Characterization of 8 putative normalization gene expression levels in MIAMI cells

In MIAMI cells expanded at low oxygen tension (3% pO_2_), real time quantitative PCR (RT-qPCR) analysis was used to determine the expression levels and relative fold difference between 8 putative normalization genes; *ACTB, B2M, EF1α, GAPDH, RPL13a, YWHAZ, UBC *and *HPRT1. HPRT1 *had the lowest expression level relative to the 7 other genes analyzed and was set to the value of 1 in order to compare with the other genes. *EF1α *(104.2 ± 0.3) and *GAPDH *(89.4 ± 1.1) had the highest relative mRNA expression levels or fold difference above *HPRT1*, followed by *RPL13a *(28.5 ± 0.3), *YWHAZ *(17.8 ± 0.4), *B2M *(11.9 ± 0.42), *UBC *(10.7 ± 0.5) and *ACTB *(8.56 ± 0.47) (Figure# 1). Alpha-2 smooth muscle aorta actin (*SMa-actin*) is another gene used for RT-qPCR normalization (Table# 1) but was not detected in MIAMI cells under expansion conditions.

**Figure 1:**
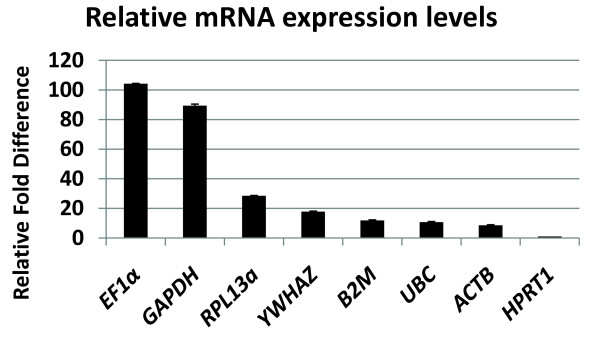
**Relative mRNA expression levels of normalization genes in MIAMI cells**. MIAMI cells expanded under normal expansion conditions (3% pO_2_) were harvested for total RNA. Real-time RT-PCR analysis was conducted using 160 nM of both forward and reverse primers with 50 ng of cDNA. Relative fold difference was calculated using the ΔΔCt method [13,22]. N = 8 independent experiments. *HPRT1 *was used as the relative control and was set to the value of 1.

The average CP standard deviation was next calculated to determine the stability of gene expression. The standard deviation of the crossing point (CP) for each gene per independent experiment (ExpSTDEV) was divided by the total number of experiments minus 1 (N-1) [ExpSTDEV/N-1]. *EF1α *(0.28) and *RPL13a *(0.29) had the lowest average CP standard deviations. *GAPDH *(1.11), which had the second highest relative expression level, had the highest average CP standard deviation between experiments. Therefore of the 8 genes tested, *EF1α *and *RPL13a *had the highest gene stability (lowest average CP standard deviation) during the expansion of MIAMI cells under low oxygen conditions, while *GAPDH *had the lowest gene stability (highest average CP standard deviation) (Figure# 2). These results validate *EF1α *and *RPL13a *as two candidate normalization genes for RT-qPCR analysis of MIAMI cells. Additionally, the high variability of *GAPDH *in MSC derived MIAMI cells is contradictory to its common use in human MSC research (Table# 1).

**Figure 2:**
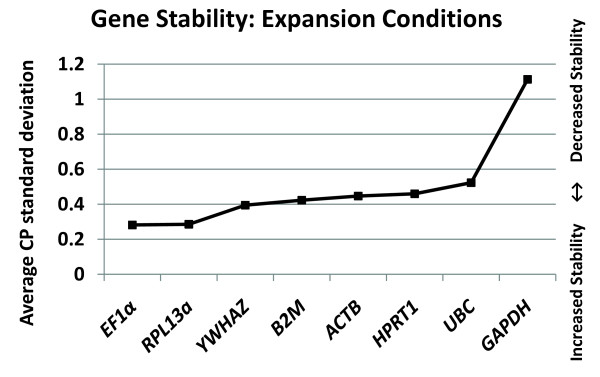
***EF1α *and *RPL13a *have the lowest average CP standard deviation**. Determination of the gene stability of 6 potential reference " housekeeping" genes during the expansion of MIAMI cells at 3% pO_2_. The gene stability was determined by comparing the average CP standard deviations for each gene between experiments. The average CP standard deviation was calculated by taking the summation of the CP standard deviations of 8 independent experiments (2-3 data points per experiment) divided by N-1. N = 8 independent experiments.

To determine the effect of gene variability on the calculation of a target genes change in expression, we used the ΔΔCP method [13]. The average CP standard deviation of the normalization gene was used to determine the theoretical deviation on the target gene, calculated as relative fold difference. The calculated theoretical effects of normalization-gene variability on the target genes fold difference are as follows: *EF1α *± 0.21, *RPL13a *± 0.22, *YWHAZ *± 0.31, *B2M *± 0.33, *ACTB *± 0.36, *HPRT1 *± 0.38, *UBC *± 0.44 and *GAPDH *± 1.16. These theoretical calculations take into account only the effect of normalization gene variability, not the additional variability of any given target gene under experimental conditions. Therefore, these data show that normalization gene variability alone can impact a target genes relative fold difference during RT-qPCR analysis, as shown with the high fold variability of *GAPDH *compared with *EF1α *and *RPL13a*.

### Stability of EF1α and RPL13a as a function of oxygen tension in MIAMI cells

*EF1α *and *RPL13a *were selected as two potential normalization "housekeeping" genes based on their high expression level and their stability during the expansion of MIAMI cells. The relative oxygen tension or partial pressure of oxygen (pO_2_) in bone marrow ranges from 1% to 7%, while in arteries the pO_2 _can reach 10-12% [14]. MIAMI cells undergo long term expansion and self-renewal at 3% pO_2, _mimicking the hypothesized *in vivo *niche environment, and require pO_2 _of 10-21% for differentiation induction [3,15]. However, heterogeneous non-selected MSC isolates are typically expanded and differentiated at 21% pO_2 _[16].

We tested the stability of *EF1α *and *RPL13a *in 1, 3, 21% pO_2 _expansion conditions. All cell cultures were expanded for at least two passages prior to RNA isolation and RT-qPCR characterization. Our results showed that the average standard CP deviation of *EF1α *remained stable irrespective of oxygen tension (1%: 0.28, 3%: 0.34, 21%: 0.33) (Figure# 3). *RPL13a *had a decreased average standard CP deviation at 1% (0.23) and 21% (0.39) compared with 3% (0.53) pO_2 _(Figure# 3). Theoretically this would produce a change in the target genes calculated relative fold differences of ± 0.21-0.26 for *EF1α *and ± 0.17-0.45 for *RPL13a *when characterizing gene expression profiles of MIAMI cells expanded under different pO_2 _expansion conditions.

**Figure 3:**
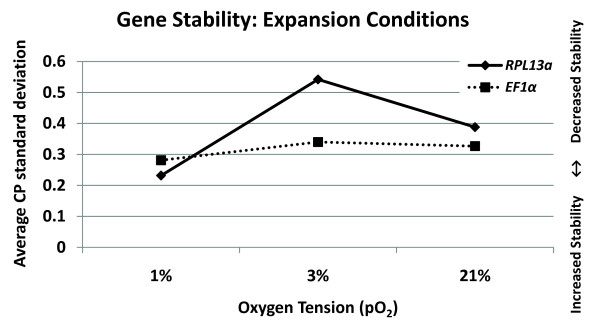
**Comparison of *EF1α *and *RPL13a *as a function of oxygen tension**. Gene stability of *EF1α *and *RPL13a *during expansion of MIAMI cells in different oxygen tensions. Gene stability was determined by comparing the average CP standard deviations for each gene between MIAMI cells expanded at 1, 3, or 21% oxygen for at least 2 passages. The average CP standard deviation was calculated by taking the summation of the CP standard deviations of 4 independent experiments (2-3 data points per experiment) divided by N-1. N = 4 independent experiments.

### Stability of EF1α and RPL13a during growth factor treatment of MIAMI cells for neural precursor enrichment

MIAMI cells are able to differentiate into cells typical of all three germ layers: endoderm, mesoderm and ectoderm [3]. In order to increase the pool of MIAMI neural precursor cells and efficiency of neurotrophin-3 (NT3) induced neuronal differentiation of MIAMI cells [17], we expanded the cells for two 5-day periods in 3% pO_2 _with 20 ng/ml each of bFGF and EGF under normal expansion conditions [3]. *EF1α *and *RPL13a *gene stability were tested under normal expansion, treated with bFGF alone or bFGF and EGF in combination. The average CP standard deviation for *EF1α *(C: 0.21, bFGF: 0.16, bFGF/EGF: 0.14) and *RPL13a *(C: 0.24, bFGF: 0.15, bFGF/EGF: 0.12) decreased with pretreatment (Figure# 4). The increased stability may be due to the formation of a more homogeneous cell population under pretreatment conditions, thereby decreasing the variability of gene expression between cell cultures and experiments.

**Figure 4:**
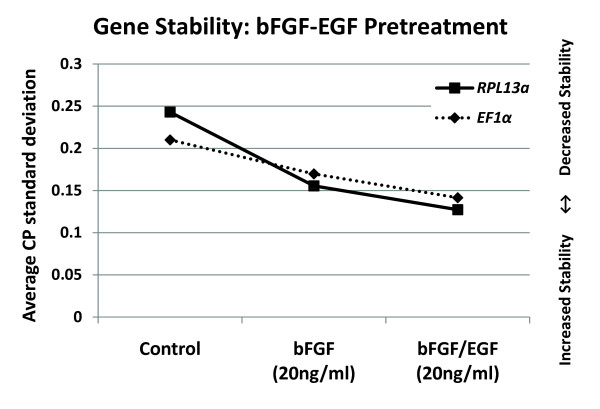
**Gene stability of *EF1α *and *RPL13a *under neural enrichment conditions**. Gene stability was determined by comparing the average CP standard deviations for each gene in MIAMI cells expanded at 3% pO_2 _with or without 20 ng/ml bFGF or bFGF/EGF treatment for 2 × 5-day periods. N = 6 independent experiments.

### Functional assessment of EF1α, RPL13a, and GAPDH as normalization genes during growth factor-induced endothelial differentiation

We determined the functional use of *EF1α *and *RPL13a *compared with the commonly used *GAPDH *as normalization genes for RT-qPCR analysis using growth factor induced endothelial differentiation of MIAMI cells. The relative fold difference of the known endothelial marker CD31 (PECAM-1: platelet endothelial cell adhesion molecule), was calculated using the ΔΔCP method. The data was normalized with *EF1α*, *RPL13a*, or *GAPDH *separately (Figure# 5A), or against the combined average after normalization against *EF1α *and *RPL13a*, or *EF1α*, *RPL13a*, and *GAPDH *together (Figure# 5B).

**Figure 5:**
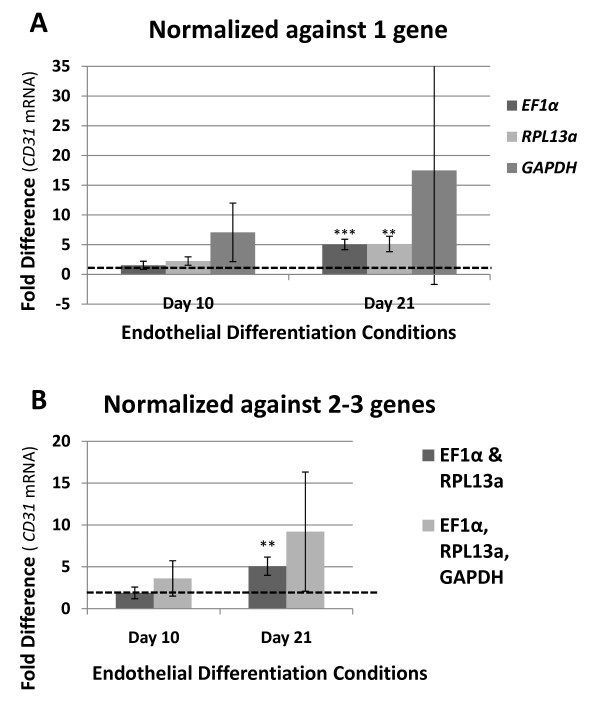
**Validation of *EF1α *and *RPL13a *as normalization genes for RT-qPCR analysis of *CD31 *mRNA expression during the endothelial differentiation of MIAMI cells**. The relative fold difference in *CD31 *mRNA expression levels during the endothelial differentiation of MIAMI cells was determined in order to test the functional use of *EF1α *and *RPL13a *as normalization genes for RT-qPCR analysis. Fold change analysis was done using the ΔΔCt method [13,22] normalizing against *EF1α*, *RPL13a*, and *GAPDH *separately or by averaging the results normalized against both *EF1α *and *RPL13a *or *EF1α*, *RPL13a*, and *GAPDH *together. Error bars are shown as standard deviation. All mRNA fold difference and statistical calculations are done relative to Day 0 which is set to the value of 1 and is denoted by the dashed line in each graph. N = 3 independent experiments.

Normalizing the RT-qPCR data against *EF1α*, *RPL13a*, or *GAPDH *individually resulted in an increase of *CD31 *mRNA expression at day ten (over day 1) of, 1.53 ± 0.68, 2.25 ± 0.71, and 7.07 ± 4.93 relative fold difference, respectively, and at day 21, 5.04 ± 0.88, 5.12 ± 1.3, and 17.49 ± 19.17 fold difference, respectively. The increase in *CD31 *at day 21 normalized against *EF1α *or *RPL13a *was statistically significant (*p *≤ 0.0005 and *p *≤ 0.0028, respectively). Due to the extremely high standard deviation, the relative fold increase of *CD31 *at day 21 normalized against *GAPDH *was not statistically significant (Figure# 5A).

We next analyzed the use of more than one normalization gene for the analysis of RT-qPCR data as recommended [8,9,18]. Using the combination of *EF1α *and *RPL13a *as normalization genes, *CD31 *had a relative fold increase at day ten of 1.89 ± 0.70, and 5.08 ± 1.09 at day 21 (*p *≤ 0.002). Using *EF1α*, *RPL13a*, and *GAPDH *together as normalization genes, *CD31 *had a fold increase at day ten of 3.62 ± 2.11, and 9.21 ± 7.12 at day 21, which was not statistically significant. In this model of endothelial differentiation of MIAMI cells, the increase of *CD31 *at day 21 normalized against both *EF1α *and *RPL13a *was statistically significant (*p *≤ 0.002). When you add in the use of *GAPDH*, the fold increase of *CD31 *at day 21 is not statistically significant (Figure# 5B). From these results we show the functionality of *EF1α *and *RPL13a *as normalization genes for the RT-qPCR analysis of MIAMI cells in this example of endothelial differentiation.

### Assessment of normalization genes used for the detection of human-specific mRNA in a rat hippocampal model of oxygen-glucose deprivation

In order to assess the role of MIAMI cells in an *ex vivo *model of global cerebral ischemia (described in Xu et al. 2002 [19]) it is important to be able to characterize the species-specific levels of mRNA expression. We created human (h) and rat (r) species-specific primer pairs to determine the change in mRNA transcript levels of human MIAMI cells injected into rat hippocampal organotypic cultures during oxygen-glucose deprivation (40 min of OGD). Primer pairs were constructed (refer to methods section) for the human target genes; stanniocalcin 1 (*hSTC1*), tumor necrosis factor-inducible protein 6 (*hTSG6*), latent transforming growth factor beta binding protein 2 (*hLTBP2*) and rat target genes; insulin growth factor 1 (*rIGF1*), insulin growth factors binding proteins 3 and 5 (*rIGFBP3 *and *rIGFBP5*). Normalization "housekeeping" genes were also constructed for rat RPL13a (*rRPL13a*), and the previously described human specific normalization genes; *hRPL13a *and *hYWHAZ*, were used for normalization of human or rat RT-qPCR data (Table# 2).

RT-qPCR analysis of human specific mRNA transcripts normalized against both *hRPL13a *and *hYWHAZ*, detected a 2.01, 2.74 and 1.62 fold increase for *hSTC1*, *hTSG6*, and *hLTBP2 *respectively (Figure# 6A). There was no detected change in *hIGF1*, *hIGFBP3*, and *hIGFBP5 *(Data not shown). Analysis of rat specific mRNA transcripts normalized against *rRPL13a *detected; no change in *rIGF1*, *rIGFBP3 *increased (1.55 ± 0.08) after the injection of MIAMI cells (compared to a media injected control), and *rIGFBP5 *was found to decrease after induction of OGD (-0.55 ± 0.26) with no change after injection of MIAMI cells. These data show the construction and functional use of human and rat species-specific primer pairs for the analysis of mRNA expression levels in an *ex vivo *cross-species animal model of global cerebral ischemia and tissue repair. This technique will allow for the future analysis of MSC, such as MIAMI cells, in animal models of tissue repair and disease.

**Figure 6:**
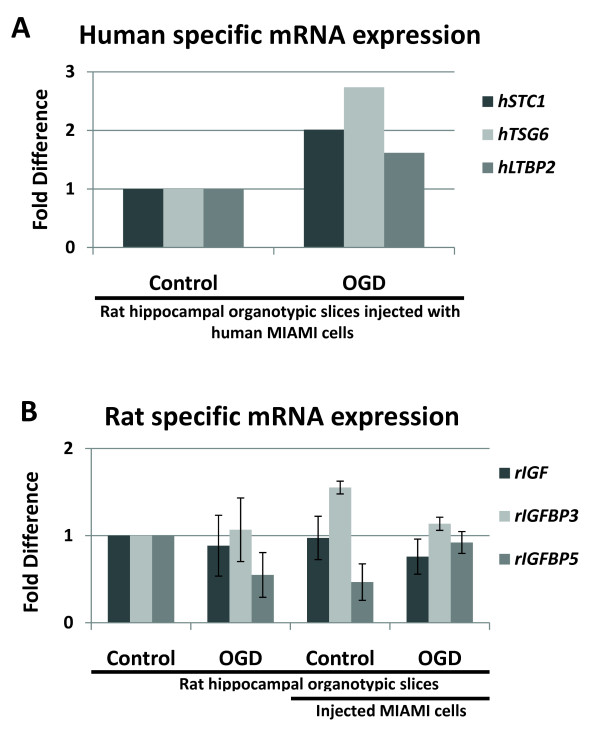
**Detection of species-specific mRNA transcripts in co-cultures of human MIAMI cells injected into rat hippocampal organotypic slices**. MIAMI cells were injected into the striatum of rat hippocampal organotypic cultures/slices (RHOS) under oxygen-glucose deprivation conditions [19]. Total RNA was isolated from each organotypic culture containing MIAMI cells. RT-qPCR analysis was completed using 5 ul of undiluted cDNA. All RT-qPCR data using species-specific primer pairs for *hSTC1*, *hTSG6*, and *hLTBP2 *were normalized against both *hRPL13a *and *hYWHAZ *(one representative experiment is shown). Rat species-specific RT-qPCR data for *rIGIF1*, *rIGFBP3*, *rIGFBP5 *were normalized against *rRPL13a*. N = 3 independent experiments.

### Comparison of 3 housekeeping genes in MIAMI cells, RS-1 cell, and MSC

To further validate the use of *RPL13a*, *EF1α*, and *GAPDH *as suitable normalization genes for RT-qPCR analysis we compared MIAMI cells with commercially available MSC (Lonza PT-2501: 21yo female) as well as an adult stem cell population, similar to MIAMI cells, derived from human MSC known as RS-1 cells (22 yo male) [7]. RT-qPCR was used to determine the level (CP) of expression for the 3 housekeeping genes, *EF1α*, *RPL13a*, and *GAPDH *in MIAMI cells, RS-1 cells, and commercially available MSC. *EF1α *and *GAPDH *had the highest expression levels (lowest CP value) in MIAMI cells expanded at 3% pO_2 _as compared with MIAMI cells, RS-1 cells and commercially available MSC expanded at 21% pO_2 _(Figure# 7A). *RPL13a *had a lower average expression level (CP: 19.28 ± 0.20) in all 3 cell types compared to *GAPDH *(CP: 15.59 ± 0.46) and *EF1α *(CP: 15.38 ± 0.23). There was no statistically significance difference between the CP values of *EF1α*, *RPL13a*, or *GAPDH *between the 3 cells types.

**Figure 7:**
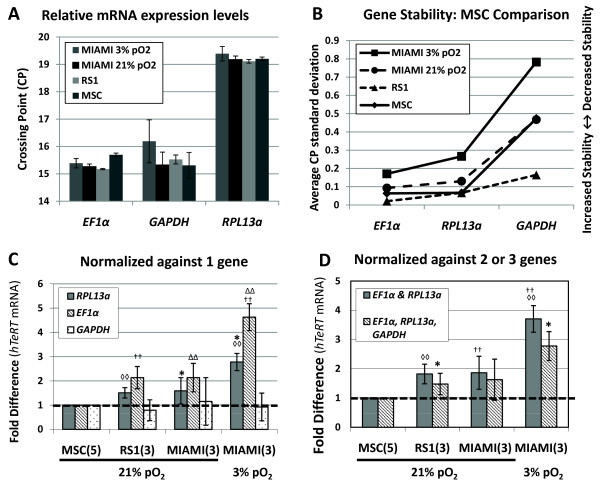
**Comparison of *hTeRT *mRNA levels in MIAMI cells, RS-1 cells, and MSC using 3 difference housekeeping genes**. MIAMI cells, RS-1 cells, and commercially available MSC were expanded at 21% pO_2 _or 3% pO_2 _from passages 1-3. Total RNA was isolated at passage 3 (MIAMI and RS-1) and passage 5 (MSC). RT-qPCR analysis was used to compare the CP levels of 3 housekeeping genes (A) as well as their average standard deviation (B). RT-qPCR analysis was used to compare human Telomerase Reverse Transcriptase (*hTeRT*) mRNA levels normalized against 1 gene (C: *EF1α*, *RPL13a*, *GAPDH*), or the average of 2-3 genes (D: *EF1α *and *RPL13a *or *EF1α*, *RPL13a *and *GAPDH*). Values are shown with standard deviation, with significant differences as p ≤ 0.01 (*) and p ≤ 0.001 (**). N = 3 independent experiments

The average CP standard deviation was used to compare the stability of expression of *EF1α*, *RPL13a*, and *GAPDH *between the 3 cell types (Figure# 7B). *EF1α *and *RPL13a *had the lowest average CP standard deviation: between MIAMI 3% pO_2 _(0.17 & 0.27), MIAMI 21% pO_2 _(0.09 & 0.13), RS-1 cells (0.02 & 0.07) and MSC (0.06 & 0.07). The average CP standard deviation of *GAPDH *was higher in MIAMI 3% pO_2 _(0.79), MIAMI 21% pO_2 _(0.47) and MSC (0.47). In RS-1 cells *GAPDH *did have a higher average CP standard deviation (0.16) compared with *EF1α *and *RPL13a*, but the increased value was not as large as seen with MIAMI cells and MSC (Figure# 7B).

In order to determine the suitability of *EF1α*, *RPL13a*, and *GAPDH *as housekeeping genes for comparison of the different bone marrow derived stromal cell populations described above, RT-qPCR analysis was used to compare the levels of human Telomerase Reverse Transcriptase (*hTeRT*), which is essential for the maintenance and propagation of telomeres.

Normalizing against *RPL13a *and *EF1α *individually, *hTeRT *mRNA levels were significantly higher in MIAMI cells expanded at 3% pO_2 _compared to RS-1 and MIAMI cells expanded at 21% pO_2_. Normalizing the RT-qPCR data against *GAPDH *alone showed no significant change in *hTeRT *levels between the 3 cell types (Figure# 7C). However using both *EF1α *and *RPL13a *in combination for normalization resulted in *hTeRT *mRNA levels significantly (p < 0.001) higher in MIAMI cells expanded at low 3% pO_2_. The use of all 3 normalization genes, *EF1α*, *RPL13a *and *GAPDH *together resulted in significance (p ≤ 0.001 vs. p ≤ 0.05) when comparing *hTeRT *mRNA fold differences between RS-1 cells and MIAMI cells expanded at 3% pO_2_. In addition, the use of all 3 genes for normalization resulted in a lower level of *hTeRT*, with no significant difference in MIAMI cells expanded at 3% pO_2 _versus 21% pO_2 _(Figure# 7D). Therefore, *GAPDH *is not a suitable RT-qPCR normalization gene either alone or in combination with *EF1α *and or *RPL13a*. Whereas the use of *EF1α *and *RPL13a *allowed for the reproducible detection of *hTeRT *mRNA levels and both produced the same relative results used alone or in combination normalization genes (Figure# 7C &7D).

## Discussion

The studies represented here show that *EF1α *and *RPL13a *are two suitable and validated housekeeping genes which can be used for the normalization of RT-qPCR data. We have shown that *EF1α *and *RPL13a *both have the lowest gene variability among 8 widely used normalization genes and can be used reproducibly in human bone marrow derived MIAMI cells under various expansion and differentiation conditions including; expansion under low and high oxygen tension, endothelial differentiation and neural precursor enrichment via treatment with bFGF/EGF. Perhaps most important is the comparison of commercially available MSC with more primitive populations of MSC, such as MIAMI and RS-1 cells. Here we have shown that *EF1α *and *RPL13a *have low gene variability in MIAMI cells as well as in RS-1 cells and in commercially available MSC, and are suitable for the comparison of gene expression between MSC derived populations, as shown with *hTeRT *analysis. In addition, species-specific primer pairs for human *RPL13a *and *YWHAZ *as well as rat *RPL13a *were found to be suitable for RT-qPCR analysis in the cross-species scenario of human MIAMI cells injected into a rat hippocampal organotypic model of ischemia. *EF1α *was not a candidate for human-rat species-specific primer pair construction due to high sequence conservation between species.

The widely used housekeeping gene, *GAPDH*, was found to have the highest level of gene instability out of 8 normalization genes tested in MIAMI cells. Moreover, we observed a decrease in significant findings when including *GAPDH *together with *RPL13a *and *EF1α *in the normalization of *CD31 *and *hTeRT *mRNA analysis of MIAMI cells, RS-1 cells, and MSC. We conclude that *GAPDH *is not a reliable housekeeping gene for the normalization of RT-qPCR data in human MSC research, contradictory to its continued usage throughout this field of research (Table# 1).

MSC derived primarily from the bone marrow have been examined extensively for their capacity to repair damaged tissues. The potential clinical applications of MSC are diverse, besides direct differentiation of the adult stem cells into the desired mature cell type; other indirect mechanisms have been identified to play important roles in the overall repair of injured tissues, treatment of autoimmune and chronic degenerative diseases. Two possible mechanisms include the production of paracrine factors or modulation of the host inflammatory response [10,20]. In order to make comparisons between heterogeneous MSC populations, as well as more homogeneous adult stem cell like MSC which are used in different laboratories throughout the world, it is important to have a standardized, reproducible set of housekeeping genes for RT-qPCR analysis. This will allow for the comparison between the *in vitro *and *in vivo *gene mRNA expression levels which would be applicable to pre-clinical and clinical analyses of the contribution of these genes to the tissue repair process and functional outcomes.

In this study we demonstrate that *EF1α *and *RPL13a *are two suitable genes for the RT-qPCR analysis and comparison of several sources of human MSC. In addition, it should be noted that this study does not and could not possibly encompass all experimental conditions or MSC populations used throughout the field of MSC research. With this in mind, it is important to note that prior to collecting and or analyzing RT-qPCR data, the housekeeping genes used for normalization must be validated.

## Conclusions

### EF1α and RPL13a are suitable genes for normalization of RT-qPCR analysis of MIAMI cells

*EF1α *and *RPL13a *have the lowest gene variability out of 8 genes tested for their use as normalization genes for RT-qPCR analysis. *GAPDH *had the highest gene variability among the 8 genes tested. *RPL13a *and *YWHAZ *were the best two genes to use for cross-species analysis of human MIAMI cells injected into a rat animal model of tissue damage, repair, and disease. *EF1α *and *RPL13a *are two suitable genes which should be used as the minimum normalization criteria for RT-qPCR analysis of commercially available MSC, RS-1 cells, and MIAMI during expansion, differentiation, and cross-species analysis.

## Methods

### MIAMI Cell Isolation

Whole bone marrow was obtained from the iliac crest of a 20 year old living male donor (Lonza Walkersville, Maryland; MIAMI #3515), and were handled and processed following the guidelines for informed consent set by the University of Miami School of Medicine Committee on the Use of Human Subjects in Research. As previously described [3], isolated whole bone marrow cells were plated at a constant density of 1 × 10^5 ^cells/cm^2 ^in DMEM-low glucose media, containing 3% fetal bovine serum (FBS, Hyclone Waltham, MA, Lot#30039), 20 mM ascorbic acid (Fluka/Sigma St. Louis, MO, #49752), an essential fatty acid mixture (Sigma St. Louis, MO; 12.9 nM arachidonic acid, (#A9673), 1.12 μM cholesterol (#C3045), 290 nM DL-alpha tocopherol-acetate (#T3376), 85.9 nM myristic acid (#M3128), 69.4 nM oleic acid (#01383), 76.5 nM palmitic acid (#P5585), 77.1 nM palmitoleic acid (P9417) and 68.9 nM stearic acid (#S4751) (modified from [21]) and antibiotics (100 U/mL penicillin, 0.1 mg/mL streptomycin) (Gibco Carlsbad, CA, #15140) on 10 ng/ml fibronectin (Sigma St. Louis, MO, #F2518) coated flasks (Nunclone Rochester, NY). Whole bone marrow cells, containing adherent and non-adherent cells, were incubated at 37°C under hypoxic conditions (3% O_2, _5% CO_2 _and 92% N_2_). Seven days later, half of the culture medium was replaced. Fourteen days after the initial plating, the non-adherent cells were removed. Pooled colonies of adherent cells were rinsed with PBS and plated at low density for expansion (100 cells/cm^2^) in 75 cm^2 ^fibronectin coated flasks.

### MIAMI Cell Culture Conditions

MIAMI cells were grown in expansion media consisting of DMEM-low glucose (as described above) in low oxygen conditions (3% O_2_, 5% CO_2 _and 92% N_2_). Media was changed every 2-3 days and the cells were detached and pelleted using trypsin (Gibco Carlsbad, CA, #25300) upon reaching ~60% confluency. Peleted cells were resuspended in media and plated in 10 ng/ml fibronectin (Sigma St Louis, MO, #F2518) coated flasks (Nunclon, Rochester, NY) at 100 cells/cm^2^. Prior to RNA isolation, adherent cells were rinsed 2× with PBS. MIAMI cells expanded for 3 passages were characterized using flow cytometry and were positive for; MHC1, CD29, CD81, CD90 and 50% positive for CD63, and negative for; MHC2, HLA-DR, CD49, CD109, CD54, CD56, CD36 (data not shown).

### RS-1 Cell Culture Conditions

Human marrow stromal cells (hMSC, Donor#7081, 22yo male) were obtained from the laboratory of Dr. Darwin Prockop, Director, Texas A&M Health Science Center College of Medicine Institute for Regenerative Medicine. Bone marrow (BM) cells were isolated from human donors according to guidelines on the Use of Human Subjects in Research as described by all commercial vendors. The hMSC were cultured in Alpha-Minimum Essential medium (αMEM) with L-glutamine, but with no ribonucleosides or deoxyribonucleosides (Invitrogen/Gibco Carlsbad, CA, #12561-056), supplemented with 16.5% FBS (Hyclone Waltham, MA, #31752), 2 mM GlutaMAX (#35050) and antibiotics (Gibco Carlsbad, CA, #15140). To enrich for RS-1 cells, hMSC(P1) were plated at 37°C under normoxic conditions (21% O_2_, 5% CO_2 _and 74% N_2_) onto 10 ng/ml fibronectin (Sigma St. Louis, MO, #F2518) coated flasks (Nunclon Rochester, NY) overnight. The cells were detached using trypsin (Gibco Carlsbad, CA #25300) and seeded at 50 cells/cm^2^. RS-1 enriched hMSCs were detached at 30-40% confluency and re-plated at low density (50 cell/cm2) [7]. RS-1 cells were harvested for RNA isolation at each passage. MSC derived RS-1 cells, passage 2, were positive for CD29, CD90, CD105 and CD73 as determined by Tulane University Center for Gene Therapy (hMSCs #7801). RS-1 cells derived in our facilities, passage 3, were positive for; MHC1, CD81, CD90, CD29 (20%), CD63 (45%) and negative for; MHC2, HLA-DR, CD49, CD109, CD54, CD56, CD36, as determined using flow cytometry analysis (data not shown).

### MSC Cell Culture Conditions

Human mesenchymal stem cells (MSC) derived from the iliac crest were purchased from Lonza (Walkersville, Maryland (PT-2501: 21yo female)). Bone marrow (BM) cells were isolated from human donors according to guidelines on the Use of Human Subjects in Research as described by all commercial vendors. The MSC were plated at 6,000 cells/cm^2 ^in DMEM-high glucose media (Gibco Carlsbad, CA, #31053) supplemented with 15% FBS (Hyclone Waltham, MA, #30039), ascorbic acid, antibiotics and essential fatty acids (as described above), and expanded at 21% O_2_, 5% CO_2 _and 92% N_2_. The entire culture media was changed every 3-4 days and the cells were detached and replated every 7 days [16]. MSC purchased from Lonza were positive for CD105, CD166, CD29, CD44, and negative for; CD14, CD34 and CD45, as determined by flow cytometry (Lonza Wlkersville, Maryland (Document # TS-PT-212-8 06/09).

### Neural Pre-treatment

Epidermal growth factor (EGF) and basic fibroblast growth factor (bFGF) treatment of MIAMI cells was performed using 20 ng/mL each of EGF (#AF-100-15) and bFGF (Peprotech Rocky Hill, NJ, #AF-100-18B) alone or in combination. The pre-treated cells were detached using trypsin and replated after day 5, followed by a second 5 day pretreatment period. Pre-treated cells were grown in expansion media under expansion conditions (3% O_2_, 5% CO_2 _and 92% N_2_). Media was changed every 2-3 days and the cells were split using trypsin (Gibco Carlsbad, CA, #25300) upon reaching ~60% confluency.

### Endothelial Differentiation

For endothelial differentiation, MIAMI cells were plated at 20,000 cells/cm^2 ^in 6 well plates (Nunclone Rochester, NY) in DMEM-low glucose media, containing 100 μM Ascorbic Acid, antibiotics, essential fatty acids, angiogenic growth factor cocktail [Sigma St. Louis, MO: 10 ng/ml bFGF, 10 ng/ml EGF, 10 ng/ml IGF; R&D Systems, Inc. Minneapolis, MN: 100 ng/ml VEGF], 100 nM Hydrocortisone, in atmosphere of 21% O_2_, 5% CO_2 _and incubated at 37°C for 21 days, with media changes every 5 days. Cells were harvested at day 10 and 21 and evaluated by RT-qPCR for the endothelial marker CD 31.

### Total RNA Sample Preparation and cDNA Synthesis

MIAMI cells were detached (Trypsin) and centrifuged to form a cell pellet. RNA was isolated using the RNAqueous^® ^-4PCR kit (Ambion Austin, TX, #AM1914) according to manufacturer's directions. Total RNA was quantified on the Nanodrop ND-1000 Spectrophotometer (Nanodrop Wilmington, DE). Reverse transcription of 2 μg total RNA to cDNA was done with random hexamer primers using the High Capacity cDNA Reverse Transcription Kit (Applied Biosystems Foster City, CA, #4368814). The cDNA was diluted 1:20 (Nuclease-Free Water: Gibco#10977-015) to a final cDNA concentration of 5 ng/μl, aliqoted, and stored at -20°C until next use. Only RNA with a 260/280 ratio between 1.9-2.0 was used for PCR analysis.

### Quantitative real-time RT-PCR (RT-qPCR)

Quantitative real-time PCR (RT-qPCR) was done using 10 μl of 1:20 diluted cDNA (50 ng) on the Mx3005P Multiplex Quantitative PCR System (Stratagene#401513) using RT-qPCR SYBR GREEN Reagents (Brilliant^® ^II SYBR^® ^Green QPCR Master Mix, Agilent Technologies) with ROX reference dye. Forward and reverse primer pairs were reconstituted in Nuclease Free Water (Gibco#10977-015). A 2 μM stock solution containing both forward and reverse primer pairs was mixed and stored at -20°C. A final concentration of 160 nM forward and reverse primer pairs was used for each RT-qPCR reaction. The cycling conditions were as follows: an initial 95°C for 10 minutes, followed by 40 cycles of 95°C for 30 sec, 58°C for 30 sec, 72°C for 15 sec. MxPro-Mx3005P v4.10 software was used to determine the CP for each amplification reaction. Results were exported to Microsoft Excel for analysis.

### Analysis of RT-qPCR data

All of the corresponding RT-qPCR data was analyzed using the ΔΔCP method [13] and normalized against one negative control, and two reference genes (housekeeping genes).

$$\text{Relative}\;\text{Fold}\;\text{Difference}=\frac{{\left({\text{E}}_{\text{target}}\right)}^{\Delta \text{CP(target gene)(Control-sample)}}}{{\left({\text{E}}_{\text{target}}\right)}^{\Delta \text{CP(reference gene)(Control-sample)}}}$$

The crossing point (CP) is defined as the point at which the fluorescence rises appreciably above the background fluorescence. The 'Fit Point Method' was used by the Mx3005P software to determine the CP for each reaction. The control sample was set to a value of "1" in all cases and error bars in the respective figures are displayed as standard deviation. The number of independent experiments is designated as "N" with 2-3 individual data points collected per experiment.

### Normalization Genes

Eight genes were tested for normalization [beta-actin (*ACTB*, NM_001101), beta-2-microglobulin (*B2M*, NM_004048), eukaryotic translational elongation factor 1 alpha (*EF1α*, NM_001402), glyceraldehyde-3-phosphate dehydrogenase (*GAPDH*, NM_002046), Hypoxanthine phosphoribosyltransferase 1 (*HPRT1*, NM_000194), ribosomal protein L13a (*RPL13a*, NM_01242), tyrosine 3-monooxygenase/tryptophan 5-monooxygenase activation protein, zeta polypeptide variant 1 & 2 (*YWHAZ*, NM_003406 & NM_145690), and ubiquitin C (*UBC*, NM_021009)]. A list of primer pair sequences used are in Table# 1.

### Determination of Primer Pair Efficiency

The determination of each genes' primer pair efficiency (E) for RT-qPCR was calculated using this equation: E = 10^(-1/m) [22]. The slope (m) was calculated by plotting the cycle number crossing point (CP) calculated during the exponential phase of the amplification plot (PxPro-Mx3005P v4.10 software) against the total cDNA concentration. Concentrations of cDNA ranged from 50-1 ng per reaction. The percent efficiency (%E) was also calculated: %E = (E-1)*100. N = 4 (2-3 data points per experiment) (Additional file # 1).

### Construction of species-specific primer pairs

In order to create species-specific primer pairs that detect only human mRNA sequences or only rat mRNA sequences within a human-rat cDNA library, the corresponding human and rat mRNA sequences must have a unique region of at least 60 bp or more. Using the human and corresponding rat FASTA mRNA sequences for *EF1α*, *RPL13a *and *YWHAZ*, we used Blast-n http://blast.ncbi.nlm.nih.gov/Blast.cgi to compare the sequences. *EF1α *had 99% sequence coverage (100% identity) between the human and rat mRNA sequences. *RPL13a *had 57% sequence coverage (87% identity) and *YWHAZ *transcript variants 1 and 2 had 92% - 63% sequence coverage (100% identity). Therefore, *RPL13a *and *YWHAZ *both were candidate human species-specific normalization genes while *EF1α *did not have a region containing a unique sequence (≥60 bp) in order to create primer pairs. Human species-specific primer pairs were constructed for the 2 normalization genes; *RPL13a *and *YWHAZ *and for 3 target genes; stanniocalcin-1 (*STC-1*), tumor necrosis factor, alpha-induced protein 6 (*TSG6*), and latent transforming growth factor binding protein 2 (*LTBP2*). NCBI Primer-BLAST http://www.ncbi.nlm.nih.gov/tools/primer-blast/ was used for primer pair sequence construction [23] using the species-unique mRNA sequences (FASTA format). Gradient PCR was used to determine optimum annealing temperature. All human and rat specific primer pairs were validated with RT-qPCR using cDNA from human MIAMI cells H3515(3) or rat hippocampal organotypic cultures either separately or in combination. All primer pairs produced 1 species-specific amplicon, with minimum off-target amplification. This was determined by the melting curve of each amplification reaction (Additional File # 2) and agarose gel electrophoresis (data not shown). Approximately 3-5 primer pairs were tested per human or rat species-specific normalization or target gene. All RT-qPCR results were normalized against a negative control, and the 2 normalization genes *hRPL13a *and *hYWHAZ *(human), or *rRPL13a *(rat). Using this same method rat specific primer pairs were also constructed for *RPL13a*, *IGF1*, *IGFBP3*, and *IGFBP5*.

### Model of ex vivo global cerebral ischemia for cross-species RT-qPCR analysis

All animal experiments were performed according to approved guidelines established by the University of Miami IACUC. The rat hippocampal organotypic slice preparation has been described in detail [19,24]. Briefly, 400 μm brain slices were obtained from rat pups of either sex between postnatal days 9 and 10. Slices were cultured for two weeks in a medium consisting of 25% heat inactivated horse serum, 50% minimal essential medium, and 25% Hank's balanced salt solution, 5.5 mg/mL D-glucose and 1 mmol/L glutamine. For ischemia we used an established model consisting of combined oxygen and glucose deprivation ([19,24]) during 40 mins. For OGD, oxygen is replaced with nitrogen and glucose with sucrose. MIAMI cells were pre-treated with bFGF and EGF (7 days: 50 ng/ml) prior to injection in the CA1 region of the hippocampus (7,500 cells/μl per injection (3 injections)). One hour after OGD induction and 24 hours after OGD total RNA was isolated from rat hippocampal organotypic slice cultures (described in [25]) with or without injected MIAMI. As described previously, 2 μg of total RNA was used for cDNA synthesis. RT-qPCR analysis was done using 5 μl of undiluted cDNA. Human and rat specific primer pairs are designated by (h) and (r) respectively (Table# 2: human specific primer pairs are designated by (*)).

### Statistical Analysis

Only data sets containing N ≥ 3 independent experiments (2-3 samples per condition per experiment) were used for statistical analysis. A One-way ANOVA followed by Tukey's post-hoc analysis was used to calculate statistical significance between conditions using GraphPad Prism version 5.00 for Windows, GraphPad Software, San Diego, CA, http://www.graphpad.com. All error bars represent standard deviation.

## Abbreviation list

MSC: Human bone marrow-derived multipotent mesenchymal stromal cells; MIAMI CELLS: marrow isolated adult multilineage inducible; RS-1: rapidly self-renewing; RT-qPCR: Quantitative real time RT-PCR; BFGF: basic fibroblast growth factor; EGF: epidermal growth factor; VEGF: vascular endothelial growth factor; OGD: oxygen-glucose deprivation;

## Competing interests

The authors declare that they have no competing interests.

## Authors' contributions

KMC: All methods involving experimental design, primer pair design, RT-qPCR, cell culture, data analysis, manuscript preparation and submission. LAG: Cell culture, endothelial differentiation of MIAMI cells, RT-qPCR. CR: Expansion of MIAMI cells at 1, 3, 21% oxygen tension, RT-qPCR. EG: Preparation of Rat hippocampal organotypic cultures and injection of MIAMI cells. APR and MAPP: Contribution of rat OGD model. PCS: Manuscript preparation. All authors have read, reviewed, edited and approved the manuscript prior to submission.

## Supplementary Material

Additional file 1 **Determination of Primer Pair Efficiency**. Table containing the calculated primer pair efficiency's for the normalization gene used during this study. Click here for file

Additional file 2 **Melting Curves for Species-Specific Primer Pairs**. This file containes the subsequent melting curves of the amplicons generated from every primer pair used for RT-qPCR analysis to determine specificity and off-target amplification. Click here for file
