# Supplementary material for: EF1α and RPL13a represent normalization genes suitable for RT-qPCR analysis of bone marrow derived mesenchymal stem cells
Source: BMC Mol Biol. 2010 Aug 17;11:61. doi: 10.1186/1471-2199-11-61 (PMC2931506; doi:10.1186/1471-2199-11-61)
Supplement: Additional file 1 — Determination of Primer Pair Efficiency. Table containing the calculated primer pair efficiency's for the normalization gene used during this study. [file 1471-2199-11-61-S1.PDF]

Primer Pair Efficiency

Gene	m	E=10^(-1/m)	% Efficiency
**EF1α**	-3.6	1.90	90%
**GAPDH**	-3.45	1.95	95%
**HPRT1**	-3.445	1.95	95%
**RPL13a**	-3.545	1.91	91%
**YWHAZ**	-3.3	2.01	101%
**UBC**	-3.55	1.91	91%

Calculation of primer pair efficiency was determined using serial dilutions of cDNA. The exponential phase amplification cycle number (CP) for each gene was plotted against the concentration of cDNA used to calculate the slope (m). The equation E=10^(-1/m) was used to calculate the efficiency for each primer pair set [21]. N=4
