# Supplementary material for: EF1α and RPL13a represent normalization genes suitable for RT-qPCR analysis of bone marrow derived mesenchymal stem cells
Source: BMC Mol Biol. 2010 Aug 17;11:61. doi: 10.1186/1471-2199-11-61 (PMC2931506; doi:10.1186/1471-2199-11-61)
Supplement: Additional file 2 — Melting Curves for Species-Specific Primer Pairs. This file containes the subsequent melting curves of the amplicons generated from every primer pair used for RT-qPCR analysis to determine specificity and off-target amplification. [file 1471-2199-11-61-S2.DOCX]

**RT-qPCR melting curves for rat and human species-specific primer pairs**


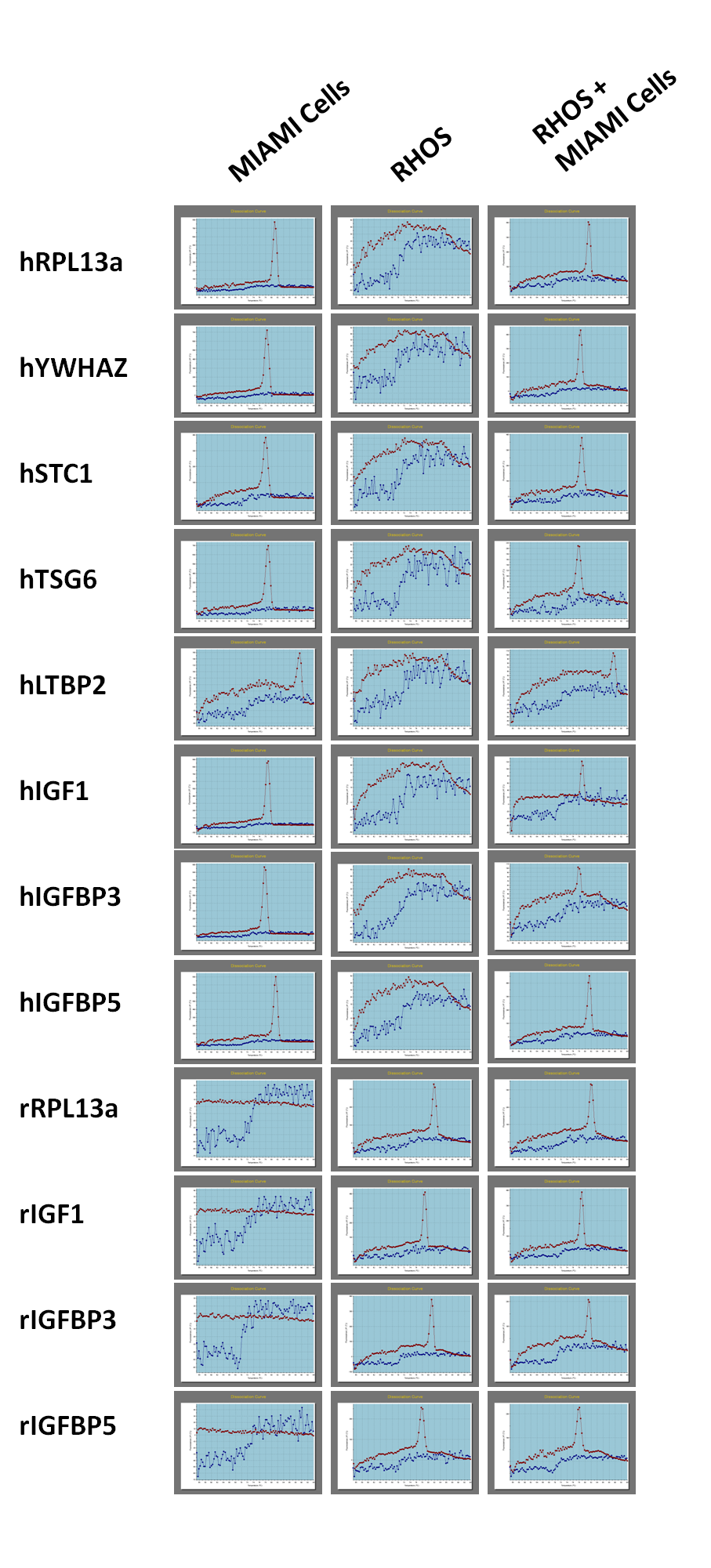


Melting curves were generated during each RT-qPCR reaction using the Mx3005P software (Stratagene). Rat and human species-specific primer pairs were tested using human derived MIAMI cells, RHOS (rat hippocampal organotypic cultures), and RHOS with injected MIAMI cells (7,000 cells in 3 injections per organotypic culture), in order to determine species specificity and non-specific off target primer pair amplification.
